# Clinical features, management, and outcome of iliopsoas abscess associated with cardiovascular disorders: a hospital-based observational case series study

**DOI:** 10.1186/s12891-019-2798-3

**Published:** 2019-10-25

**Authors:** Sung-Yuan Hu, Ming-Shun Hsieh, Yao-Tien Chang, Chih-Che Huang, Che-An Tsai, Chung-Lin Tsai, Chiann-Yi Hsu, Chia-Hui Shen, Yan-Zin Chang

**Affiliations:** 1 Institute of Medicine, Chung Shan Medical University, No. 110, Sec. 1, Jianguo N. Rd, Taichung City, 40201 Taiwan; 2 School of Medicine, Chung Shan Medical University, Taichung City, Taiwan; 3 Department of Emergency Medicine, Taichung Veterans General Hospital, 1650 Taiwan Boulevard Sect. 4, Taichung City, 40705 Taiwan; 4 Department of Nursing, College of Health, National Taichung University of Technology and Science, Taichung City, Taiwan; 5 Department of Nursing, Central Taichung University of Technology and Science, Taichung City, Taiwan; 6 School of Medicine, National Yang-Min University, Taipei City, Taiwan; 7 Department of Nursing, Jen-Teh Junior College of Medicine, Nursing and Management, Miao-Li County, Taiwan; 8 Department of Emergency Medicine, Taipei Veterans General Hospital, Tao-Yuan Branch, No. 100, Sec. 3, Cheng-Kung Road, Taoyuan, 330 Taiwan; 9 Department of Emergency Medicine, Taipei Veterans General Hospital, Taipei City, Taiwan; 10 Institute of Occupational Medicine and Industrial Hygiene, College of Public Health, National Taiwan University, Taipei City, Taiwan; 11 Department of Medical Research, Taichung Veterans General Hospital, Taichung City, Taiwan; 12 Division of Infectious Disease, Department of Internal Medicine, Taichung Veterans General Hospital, Taichung City, Taiwan; 13 Division of Cardiovascular Surgery, Department of Surgery, Taichung Veterans General Hospital, Taichung City, Taiwan; 14 Department of Clinical Laboratory, Drug Testing Center, Chung-Shan Medical University Hospital, Taichung, Taiwan

**Keywords:** Computed tomography (CT), Stent-graft/Endograft infection, Infective endocarditis, Iliopsoas abscess (IPA), Mycotic aneurysm

## Abstract

**Background:**

Iliopsoas abscess (IPA) is a rare clinical entity and is difficult to diagnose due to its insidious onset and nonspecific symptoms. The association between IPA and cardiovascular disorders (CVD) has been rarely reported. Computed tomographic (CT) scan can provide a definitive diagnosis of IPA and associated foci of adjacent structures. IPA is a life-threatening condition, especially when associated with CVD.

**Materials and methods:**

We conducted a hospital-based observational study of IPA associated with CVD. Data were collected from the electronic clinical database of Taichung Veterans General Hospital (1520-bed tertiary referral hospital in central Taiwan) between July 2007 and December 2017. The diagnosis of IPA associated with CVD was confirmed by classical findings on CT and transesophageal echocardiography with compatible clinical presentation and cultures from pus/tissue and blood.

**Results:**

Fifteen patients of IPA associated with CVD were studied. They included 12 males (80%) and 3 females (20%), with a mean age 63.2 ± 16.9 years (31–85 years). CVD included stent-graft/endograft infection of abdominal aortic aneurysm (AAA) (40%), primary mycotic AAA (33.3%), and infective endocarditis (26.7%). *Staphylococcus aureus* is the most common microorganism in pus/tissue cultures (*n* = 3, 37.5%) and in blood cultures (*n* = 6, 40%). The average length of hospital stay was 33.1 ± 20.5 days (range, 3–81 days; median, 33 days). Hospital stay lasted 42.6 ± 19.2 days in the survival group and 19.0 ± 14.1 days (*P* = 0.018) in the non-survival group. Incidence of patients staying in the intensive care unit (ICU) with intubation > 3 days was 33% in the survival group and 100% (*P* = 0.028) in the non-survival group. Intra-hospital mortality rate was 40%. Poor prognostic factors in the non-survival group were hypoalbuminemia, hyponatremia, involved disc/vertebral body and/or epidural abscess, and ICU stay with intubation > 3 days. Cumulative survival rate was 25% under conservative treatments and 66.3% under aggressive treatments (*P* = 0.038).

**Conclusion:**

Due to high mortality rates, clinicians should keep a high suspicion index for IPA associated with CVD through clinical presentation, physical examination, and imaging study. Timely empiric antibiotics for common bacteria, drainage for IPA, endovascular repair, or vascular reconstruction by graft replacement or bypass with intensive care should be mandatory to shorten the hospital stay, reduce medical costs, and lower mortality rate.

## Background

Iliopsoas abscess (IPA) presents an infection with purulent materials appearing within iliopsoas muscle components. It is a rare clinical entity and difficult to diagnose due to its insidious onset and nonspecific symptoms. IPA is classified into primary and secondary types depending on the origin of infectious focus. Primary IPA is originated from an infection of distant source spread through hematological or lymphatic routes. Secondary IPA is an infectious process involving adjacent structures via direct invasion. *Staphylococcus aureus* is the most common microorganism in primary IPA and secondary IPA related to skeletal muscular infections. Enteric bacteria, such as *Escherichia coli*, *Klebsiella pneumoniae* and *Salmonella*, are the leading microorganisms in secondary IPA caused by intraabdominal infection, and often with mixed polymicrobial presentations [1–4]. Little is known on the association between IPA and CVD. Cardiovascular disorders include primary aortic mycotic aneurysm, endograft infection of the aorta, and infective endocarditis [5–8]. Computed tomography (CT) provides a rather definitive diagnosis of IPA and associated focus of adjacent structure [1–8]. Clinical managements should include empiric antibiotics, percutaneous drainage (PCD), and open surgery for to remove the infectious structures involved [5–8]. When delayed in diagnosis, IPA is a life-threatening condition. The mortality is higher for secondary IPA than for primary IPA, especially those associated with CVD. The mortality reaches up to 100% if the condition is left untreated [7]. Here, we carried out a hospital-based observational case series study of IPA associated with CVD at a referral medical center in central Taiwan.

## Materials and methods

This study was approved by the institutional review board of Taichung Veterans General Hospital (No. CE18102A). Data were collected from the electronic database of Taichung Veterans General Hospital, a 1520-bed tertiary referral hospital in central Taiwan. Patients were selected for this study based on the ICD-10 codes, including K68.12 (iliopsoas abscess, psoas muscle abscess), I71.4 (abdominal aortic aneurysm), I71.6 (thoracoabdominal aortic aneurysm), I71.9 (aortic aneurysm), I72.9 (mycotic aneurysm), I33.9 (acute endocarditis), and I38 (endocarditis). IPA associated with CVD (primary aortic mycotic aneurysm, stent-graft/endograft infection of the aorta, and infective endocarditis) was confirmed in diagnosis by the classical findings on CT with contrast media and transesophageal echocardiography (TEE), compatible with clinical presentation, laboratory investigations, and cultures of pus/tissue and blood. Typical CT images of IPA associated with CVD included primary mycotic abdominal aortic aneurysm (AAA) and stent-graft/endograft infection of AAA with abscess within the iliopsoas muscle with or without gas. All CT images were reviewed by the primary study investigator and a radiologist. Infective endocarditis with vegetation was confirmed through TEE by a cardiologist.

The primary outcome of this study was the intra-hospital mortality of patients who had been treated for IPA associated with CVD and discharged from hospital with improvement of laboratory data and clinical conditions. During hospitalization, respiratory failure with intubation admitted to ICU with intubation > 3 days was defined as a clinical complication of IPA associated with CVD. We defined “successful treatment” as the improvement in clinical conditions, in follow-up imaging, and discharged alive after treatment with PCD, surgery, or antibiotics alone. “Failed treatment” was defined as mortality during hospitalization or deterioration of clinical conditions with accompanying non-decremental changes in follow-up imaging, all of which had necessitated another advanced treatment modality (i.e., antibiotics plus PCD, antibiotics plus surgery, or PCD followed by surgery). Patients received a minimum follow-up period of 1 year. Excluded cases were those ≤18 years of age (according to the law in Taiwan, informed consent must be signed by their parents), or had an incomplete treatment course.

Fifteen patients of IPA associated with CVD admitted to our hospital between July 2007 and December 2017 were included in this study. Demographic data, laboratory investigations, etiological pathogens, infectious origins, management approaches, clinical process, and treatment outcome were coded for further statistical analyses. Continuous variables were presented as mean ± SD, and categorical variables as numbers and percentages. Further comparisons were performed for continuous variables using Mann–Whitney U test and for categorical variables using chi-square test or Fisher’s exact test. *P* values < 0.05 were considered statistically significant. Analyses were performed using the Statistical Package for the Social Science (IBM SPSS version 22.0; International Business Machines Corp, New York, USA).

## Results

### Clinical features

Fifteen patients of IPA associated with CVD were enrolled, including 12 males (80%) and 3 females (20%), aged 63.2 ± 16.9 years (range, 31–85 years). CVD included stent-graft/endograft infection of AAA (6 patients, 40%), primary mycotic AAA (5 patients, 33.3%), and infective endocarditis (4 patients, 26.7%).

Nine patients (60%) had respiratory failure that required intubation and stay in ICU for > 3 days, and 6 patients (40%) had respiratory failure with intubation but the stay in ICU for ≤3 days. Incidence of patients staying in the ICU with intubation for over 3 days was 33% for the survival group and 100% (*P* = 0.028) for the non-survival group. The ICU stay with intubation for ≤3 days (67% vs. 0%, *P* = 0.028) was a good prognostic factor for survival group. The average length of hospital stay was 33.1 ± 20.5 days (range, 3–81 days; median, 33 days). The hospital stay was 42.6 ± 19.2 days for the survival group, and 19.0 ± 14.1 days (*P* = 0.018) for the non-survival group. The overall intra-hospital mortality rate was 40%. The demographic characteristics were summarized in Table 1.

**Table 1:** General demographics of 15 patients with iliopsoas abscess associated with cardiovascular disorders

	Clinical outcome				Total (*n*, %; mean ± SD)		*P* value
	Alive (*n*, %; mean ± SD)		Death (*n*, %; mean ± SD)				
Gender							0.525
Male	8	(89%)	4	(67%)	12	(80%)	
Female	1	(11%)	2	(33%)	3	(20%)	
Age^#^	63.8	±16.4	62.3	±19.3	63.2	±16.9	1.000
Hospital stay (days)	42.6	±19.2	19.0	±14.1	33.1	±20.5	0.018*
Complicated condition							0.028*
Intubation with intensive care unit (ICU) stay ≤3 days	6	(67%)	0	(0%)	6	(40%)	
Intubation with ICU stay > 3 days	3	(33%)	6	(100%)	9	(60%)	
Lactatemia (Lactate > 12 mg/dl)	7	(88%)	4	(80%)	11	(85%)	1.000
Hypoalbuminemia (Albumin < 2.5 g/dl)	2	(22%)	6	(100%)	8	(53%)	0.007**
Anemia (Hemoglobin < 10 g/dl)	4	(44%)	3	(50%)	7	(47%)	1.000
Leukocytosis (White blood cells > 12,000/mm^3^)	6	(67%)	4	(67%)	10	(67%)	1.000
Left shift phenomenon (Neutrophils > 80%)	6	(75%)	4	(67%)	10	(71%)	1.000
Bandemia	2	(22%)	2	(33%)	4	(27%)	1.000
Thrombocytopenia (Thrombocytes < 140,000/mm^3^)	2	(22%)	2	(33%)	4	(27%)	1.000
Hyponatremia (Sodium < 135 mEq/l)	7	(78%)	5	(83%)	12	(80%)	1.000
Hyperkalemia (Potassium > 5.3 mEq/l)	0	(0%)	1	(17%)	1	(7%)	0.400
Hypokalemia (Potassium < 3.5 mEq/l)	0	(0%)	1	(17%)	1	(7%)	0.400
Impaired renal function							
Blood urea nitrogen (BUN > 25 mg/dl)	4	(44%)	2	(33%)	6	(40%)	1.000
Creatinine (Cr > 1.4 mg/dl)	4	(44%)	3	(50%)	7	(47%)	1.000
BUN/Cr (> 20)	3	(33%)	3	(50%)	6	(40%)	0.622
Impaired liver function (GPT > 50 U/l or GOT > 40 U/l)	4	(44%)	1	(17%)	5	(33%)	0.580
Hyperglycemia (Blood glucose > 200 mg/dl)	3	(33%)	4	(67%)	7	(47%)	0.315
Systolic blood pressure (mmHg)^#^	113.7	±14.8	118.2	±20.9	115.5	±16.9	0.768
Diastolic blood pressure (mmHg)^#^	68.4	±10.7	73.2	±14.0	70.3	±11.9	0.479
Heart rate (beats/min)^#^	105.8	±19.7	103.8	±17.4	105.0	±18.2	0.679
Body temperature (°C)^#^	37.0	±1.5	37.2	±0.7	37.1	±1.2	0.407
Respiratory rate (breaths/min)^#^	18.6	±1.3	19.7	±2.0	19.0	±1.6	0.346
Systemic inflammatory response syndrome	7	(78%)	5	(83%)	12	(80%)	1.000
Infectious source ^c^							0.870
Primary mycotic abdominal aortic aneurysm	4	(44%)	2	(33%)	6	(40%)	
Stent-graft/endograft infection	3	(33%)	2	(33%)	5	(33%)	
Infective endocarditis	2	(22%)	2	(33%)	4	(27%)	
Clinical treatment							0.235
Antibiotics only	1	(11%)	3	(50%)	4	(27%)	
Drainage/Surgery	8	(89%)	3	(50%)	11	(73%)	
Blood culture (*n* = 9 vs. 5)							0.580
Monomicrobial (Gram-positive and Gram-negative)	7	(78%)	3	(60%)	10	(71%)	
Polymicrobial	2	(22%)	2	(40%)	4	(29%)	
Pus/Tissue culture (*n* = 4 vs. 3)							0.429
Monomicrobial (Gram-positive only)	2	(50%)	0	(0%)	2	(29%)	
Polymicrobial	2	(50%)	3	(100%)	5	(71%)	
Treatment at other rural hospitals	2	(22%)	2	(33%)	4	(27%)	1.000
Maximum diameter of iliopsoas abscess (IPA)^#^	3.7	±1.4	4.5	±2.0	4.0	±1.6	0.480
Maximum diameter of IPA > 3 cm	5	(56%)	4	(67%)	9	(60%)	1.000
Gas-forming	2	(22%)	3	(50%)	5	(33%)	0.329
Involvement ^c^							0.683
Right	3	(33%)	1	(17%)	4	(27%)	
Left	2	(22%)	1	(17%)	3	(20%)	
Bilateral	4	(44%)	4	(67%)	8	(53%)	
Multiple lobulated	9	(100%)	6	(100%)	15	(100%)	–
Discitis/Paraspinal/Epidural abscess	0	(0%)	3	(50%)	3	(20%)	0.044*
Receiving transesophageal echocardiography (TEE)	6	(67%)	3	(50%)	9	(60%)	0.622
Infective endocarditis found on TEE	2	(22%)	2	(33%)	4	(27%)	1.000
Recurrence of IPA	3	(33%)	0	(0%)	3	(20%)	0.229
Tool for follow up							0.329
Computed tomographic scan	7	(78%)	3	(50%)	10	(67%)	
No mentioned or Unknown	2	(22%)	3	(50%)	5	(33%)	
Hypertension	5	(56%)	3	(50%)	8	(53%)	1.000
Diabetes mellitus	3	(33%)	4	(67%)	7	(47%)	0.315
Cerebrovascular accident	2	(22%)	2	(33%)	4	(27%)	1.000
Bed-ridden status	1	(11%)	3	(50%)	4	(27%)	0.235
Malignancy	2	(22%)	1	(17%)	3	(20%)	1.000
Chronic kidney disease	3	(33%)	0	(0%)	3	(20%)	0.229
Alcoholism	2	(22%)	1	(17%)	3	(20%)	1.000
Hemodialysis	1	(11%)	0	(0%)	1	(7%)	1.000
Intravenous drug abuse	1	(11%)	1	(17%)	2	(13%)	1.000

^#^Mann-Whitney U test. Fisher’s exact test. ^c^Chi-Square test. *p < 0.05, **p < 0.01

Continuous data were expressed median and IQR

Categorical data were expressed number and percentage

### Laboratory investigations

The laboratory investigations were summarized in Table 2. Twelve patients (80%) had systemic inflammatory response syndrome with 2 or more of the following variables: (1) fever of > 38 °C (100.4 °F) or < 36 °C (96.8 °F); (2) heart rate of > 90 beats/min; (3) respiratory rate of > 20 breaths/min or arterial CO_2_ tension (P_a_CO_2_) of < 32 mmHg; (4) abnormal white blood cells (> 12,000/mm3 or < 4000/mm3 or > 10% immature [band] forms). We found that hypoalbuminemia (2.9 ± 0.5 vs. 2.2 ± 0.3, *P* = 0.009) and hyponatremia (132.6 ± 2.6 vs. 128.8 ± 3.6, *P* = 0.032) were poor prognostic factors for the non-survival group.

**Table 2:** Laboratory investigations of 15 patients with iliopsoas abscess associated with cardiovascular disorders

	Clinical outcome				Total (*n* = 15; mean ± SD)		*P* value
	Alive (*n* = 9; mean ± SD)		Death (*n* = 6; mean ± SD)				
White blood cells (× 10^3^/mm^3^)^#^	14.1	±5.5	16.8	±9.1	15.2	±7.0	0.906
Neutrophils (%)^#^	84.3	±10.5	81.9	±13.5	83.3	±11.4	0.860
Band (%)^#^	2.6	±5.1	0.8	±1.6	1.9	±4.1	0.880
Hemoglobin (g/dl)^#^	10.9	±2.2	11.1	±3.1	11.0	±2.5	0.860
Thrombocytes (×10^3^/mm^3^)^#^	241.4	±191.8	188.0	±114.5	220.1	±162.6	0.480
Albumin (g/dl)^#^	2.9	±0.5	2.2	±0.3	2.6	±0.6	0.009**
Alkaline phosphatase (U/l)^#^	189.0	±83.3	281.7	±195.2	226.1	±140.7	0.316
Glutamic oxaloacetic transaminase (U/l)^#^	38.7	±27.8	32.8	±14.5	36.6	±23.4	0.640
Glutamic pyruvic transaminase (U/l)^#^	45.2	±33.4	38.5	±18.7	42.5	±27.8	0.953
Lactic dehydrogenase (U/l)^#^	260.4	±87.0	246.2	±117.3	255.4	±94.6	0.739
C-reactive protein (mg/dl)^#^	19.2	±9.7	15.5	±5.4	17.7	±8.2	0.409
Glucose (mg/dl)^#^	192.0	±85.6	223.0	±126.5	204.4	±100.8	1.000
Lactate (mg/dl)^#^	26.2	±25.4	38.4	±45.4	30.9	±33.2	0.558
Sodium (mEq/l)^#^	132.6	±2.6	128.8	±3.6	131.1	±3.5	0.032*
Potassium (mEq/l)^#^	4.0	±0.4	4.7	±0.9	4.3	±0.7	0.058
Blood urea nitrogen (mg/dl)^#^	44.0	±59.2	23.8	±9.2	35.9	±46.2	0.953
Creatinine (mg/dl)^#^	2.1	±2.3	1.4	±0.4	1.8	±1.8	0.723
pH^#^	7.4	±0.1	7.5	±0.1	7.4	±0.1	0.637
P_a_CO_2_ (mmHg)^#^	35.1	±9.7	32.8	±8.5	34.2	±9.0	0.637
HCO_3_^−^ (mEq/l)^#^	22.4	±6.1	22.8	±7.0	22.6	±6.2	0.814

^#^Mann-Whitney U test. Fisher’s exact test. ^c^Chi-Square test. *p < 0.05, **p < 0.01

Continuous data were expressed median and IQR

Categorical data were expressed number and percentage

### Etiological pathogens

Results of pus/tissue cultures showed monomicrobial infection in 2 patients, polymicrobial infection in 5 patients, and no growth in 1 patient. No culture of pus/tissue was available in 7 patients due to treatment with antibiotics only and prior treatment at other rural hospitals. Results of blood culture showed monomicrobial infection in 10 patients (5 with Gram-positive and 5 with Gram-negative), polymicrobial infection in 4 patients, and no growth in 1 patient. The results of culturing pus/tissue and blood were summarized in Table 3.

**Table 3:** Microorganisms isolated from 15 patients of iliopsoas abscess associated with cardiovascular disorders

Microorganisms	Culture of pus/tissue (n, %)	Culture of blood (n, %)
Gram-positive	5 (33.3%)	9 (60%)
*Staphylococcus aureus*	3 (20%)	6 (40%)
*Streptococcus mitis* group	1 (6.7%)	1 (6.7%)
*Cutibacterium acnes* (*Propionibacterium acnes*)**^**	1 (6.7%)	0 (0%)
*Bacillus cereus*	0 (0%)	1 (6.7%)
*Listeria monocytogenes*^**%**^	0 (0%)	1 (6.7%)
Gram-negative	5 (33.3%)	8 (53.3%)
*Salmonella*^**$**^	1 (6.7%)	4 (26.7%)
*Acinetobacter baumannii*	1 (6.7%)	0 (0%)
*Escherichia coli*	1 (6.7%)	1 (6.7%)
*Klebsiella pneumoniae*	1 (6.7%)	1 (6.7%)
*Prevotella* sp.^ *	1 (6.7%)	0 (0%)
*Pseudomonas aeruginosa*	0 (0%)	1 (6.7%)
*Veillonella* sp.**^**	0 (0%)	1 (6.7%)
Fungus	2 (13.3%)	1 (6.7%)
*Candida*^**#**^	2 (13.3%)	1 (6.7%)

1. **^** presented anaerobic; ^**%**^ presented facultative anaerobic

2. ^**$**^ including *Salmonella enterica* serovar Typhimurium and *Salmonella enterica* serovar Enteritidis”; * including *P. melaninogenica* and *P. oralis*; ^**#**^ including *C. tropicalis*, *C. albicans*, and unclassified *Candida* sp.

3. Polymicrobial infections of pus/tissue and blood were found in 5 and 4 patients, respectively

### Imaging studies

Of 15 patients, all received CT scan for a definitive diagnosis of IPA associated with CVD and 9 patients underwent TEE. Of 15 patients, depicted on their CT, maximum diameter of objects > 3 cm was found in 9 patients (60%), bilateral involvement in 8 patients (53.3%), and gas-formation in 5 patients (33.3%). Involving disc/vertebral body and/or epidural abscess detected by CT was 0% in the survival group, and 50% (*P* = 0.044) in the non-survival group. Through TEE in 4 patients, we detected infective endocarditis that involved aortic (*n* = 2) and tricuspid (*n* = 2) valves.

### Management and outcomes

**Fig. 1:**
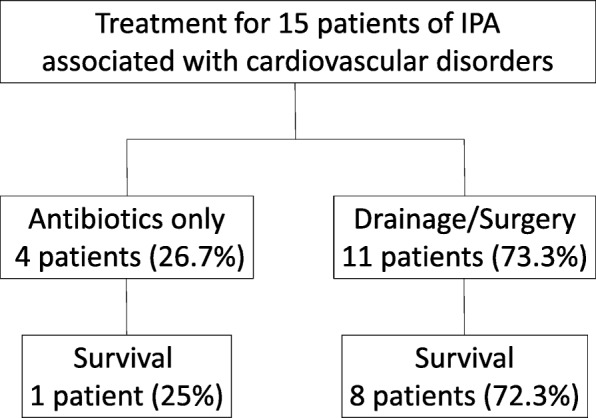
Treatment algorithm for 15 patients of iliopsoas abscess associated with cardiovascular disorders

**Fig. 2:**
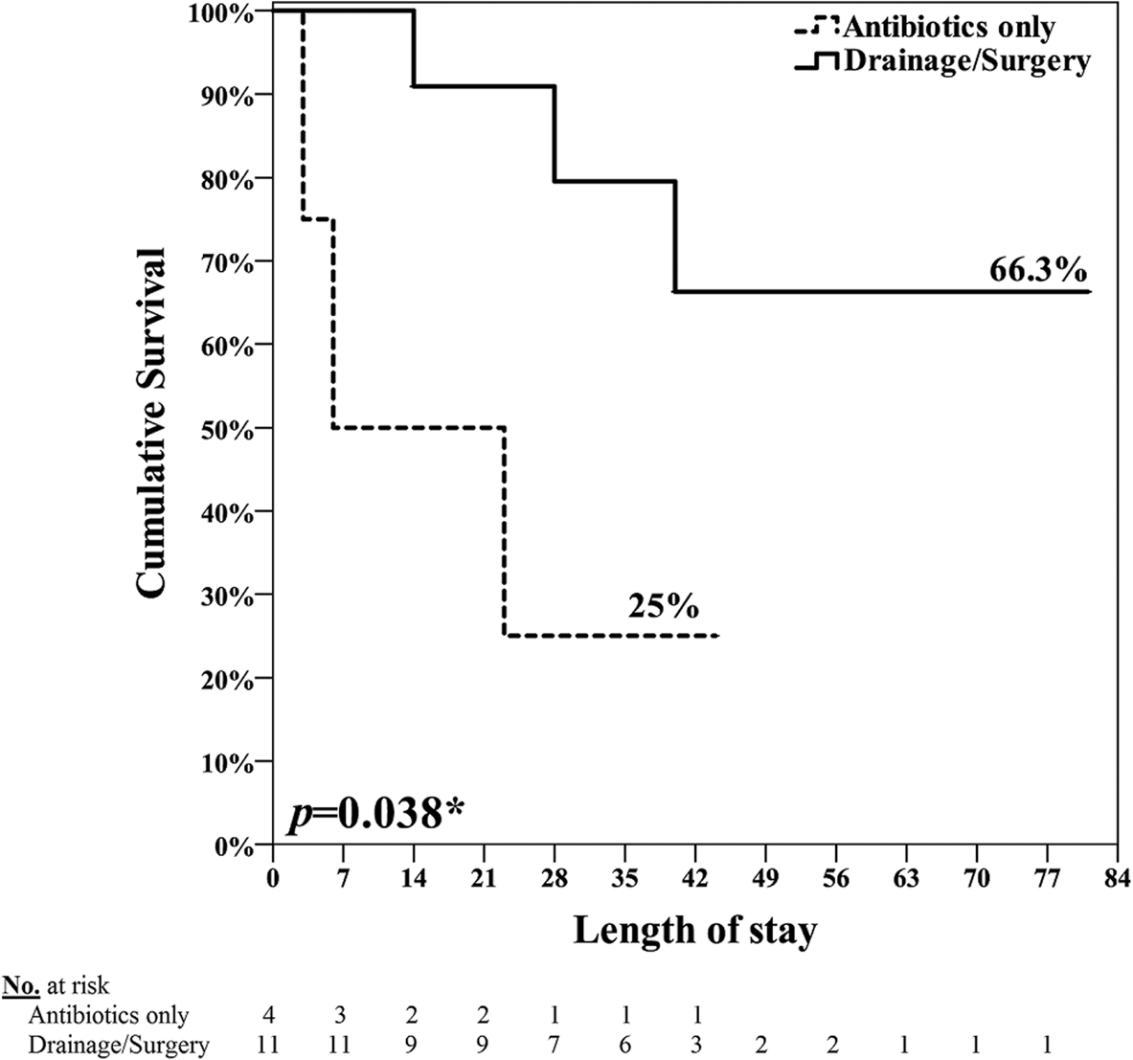
Cumulative survival rate in conservative (antibiotics only) and aggressive (drainage/surgery) groups

Our approach algorithm of IPA associated with CVD was shown in Fig. 1. On antibiotics-based treatment, PCD or surgical drainage and combination with endovascular graft or surgical intervention of graft replacement and bypass produced good outcome compared to treatment of antibiotics alone. Conservative treatment with antibiotics was prescribed to patients due to severe infection complicated with unstable hemodynamics or bleeding tendency (thrombocytopenia or prolong PT/APTT), who were not suitable for PCD or surgical intervention. Aggressive management was carried out for vascular repair and eradiation of infection, including endovascular or surgical repairing of the primary mycotic AAA and stent-graft/endograft infection, and PCD or surgical drainage of IPA according to the clinical conditions. Cumulative survival rate for conservative treatment was 25% and for aggressive management was 66.3% (*P* = 0.038) (Fig. 2).

## Discussion

### Comparison with the most recent studies

Here, we found that CVD included infected vascular graft (40%), primary mycotic AAA (33.3%), and endocarditis (26.7%). These results were similar to those by Ouellette et al., on 109 patients of secondary IPA. They reported 15 cases (13.8%) of IPA caused by CVD, that included infected vascular graft (6, 40.0%), infected catheter (5, 33.3%), endocarditis (3, 20.0%), and infected fistula (1, 6.7%) [9]. We found the most common microorganism of blood culture beings Gram-positive cocci (9, 60%), like staphylococci (6, 40%) and streptococci (1, 6.7%). Our results were similar to those by Nakamura et al., who reported Gram-positive cocci (63, 50%) in 126 patients with psoas abscess and pyogenic spondylitis, including staphylococci (51, 40%) and streptococci (8, 6.3%) [10]. For IPA caused by CVD, we recommended here treatment with PCD or surgical drainage and combination with graft replacement to eradicate infection foci. Clinical management with PCD and appropriate antibiotic therapy was safe and effective with shorter hospital stay for IPA. Open surgical intervention might be warranted if there was an underlying pathology [9–11]. Risk factors associated with mortality in IPA caused by CVD were found in our present study to include hypoalbuminemia, hyponatremia, involving disc/vertebral body and/or epidural abscess, and ICU stay with intubation > 3 days. Nakamura et al. also reported that the mortality was related to hypotension, hypoalbuminemia, liver failure, and renal dysfunction in patients with psoas abscess and pyogenic spondylitis [10]. Intra-hospital mortality rate was 40% in our present report. If patients had cardiovascular disorders, there was a higher risk of mortality in those with psoas abscess or pyogenic spondylitis as reported by Nakamura et al. [10]. We reviewed the following scientific reports regarding the anatomy of iliopsoas muscle, clinical etiology, microbiology, management, and outcome of IPA caused by CVD.

### Anatomy of the iliopsoas muscle

Psoas muscle is a long fusiform muscle located along the low thoracic, lumbar, and first sacral region of the vertebral column and brim of the lesser pelvis. It joins the iliacus muscle and together forms the iliopsoas muscle (IPM). It lies in close proximity to the sigmoid colon, appendix, jejunum, ureters, abdominal aorta, kidneys, pancreas, spine, and iliac lymph nodes. Hence any infectious process of these organs could extend into the IPM. The abundant blood supply and lymphatic drain of IPM may predispose itself to hematogenous or lymphatic spread from an occult infection at a distant site [1–3].

### Definition of iliopsoas abscess

Iliopsoas abscess (IPA) has been traditionally considered a rare infection with purulent materials developed within the IPM compartment. IPA was first described by Mynter in 1881 [1, 2, 4]. Pyogenic IPA is divided into two types: primary and secondary. Primary IPA develops most likely secondary to Staphylococcal bacteremia from an occult infection in the body owing to the rich vascular supply and lymphatic drain of IPM. It is predominantly seen in younger patients and in the developing and tropical countries (Asia and Africa). Secondary IPA is caused by contiguous spread from an infectious process of the adjacent structures (gastrointestinal tract, urinary tract, vessel, and spine). The condition is commonly found in Europe and North America, with the mixed enteric bacteria being the major microorganisms involved [1, 2, 5–13]. Primary and secondary IPA occurred in a frequency ratio of 11 to 89%, respectively [13].

### Mechanisms and risk factors of iliopsoas abscess and cardiovascular disorders

Spondylodiscitis associated with the epidural, paraspinal, and psoas abscesses are caused by hematogenous or lymphatic spread, and a direct extension from the adjacent focus like infective endocarditis and infected aortic aneurysm or endograft [14–18]. Patients with IPA should be investigated for infective endocarditis, particularly caused by *Staphylococcus aureus* [16]. Emphysematous IPA is an unusual complication of mycotic AAA. Progressive inflammatory process extending beyond the arterial wall with a point of leakage in the arterial adventitia into the surrounding IPM may result in a localized abscess with gas collection and may penetrate the arterial adventitia leading to retroperitoneal hemorrhage and sudden death [18–20]. The possible mechanisms of mycotic aneurysm include bacteremia with septic emboli, which enter the vasa vasorum and cause vessel-wall infection leading to aneurysm formation, systemic pathogens inoculating in the existing aortic aneurysm, and aneurysm formation from an extravascular source or adjacent tissues [19, 21, 22].

The predisposing factors for the development of aortic infective aneurysms are old age (≥65 years), pre-existing aneurysm, atherosclerotic disease, diabetes mellitus (DM), hypertension, hyperlipidemia, rheumatoid arthritis, malignancy, and acquired immune deficiency syndrome. The intimal damage and atherosclerosis promote the development of mycotic aneurysm [18, 21]. DM (64%) is the dominant predisposing or associated factor of IPA. Autonomic neuropathy of small bowel reduces intestinal motility in diabetic patients, raises intestinal transit time and therefore increases the risk of Salmonellosis associated with IPA and infected aortic aneurysm [7, 23]. In our present study, the incidence of old age (≥65 years) was 80%, hypertension 53.3%, DM 46.7%, and Salmonellosis 26.7%.

### Incidence and the association between iliopsoas abscess and cardiovascular disorders

IPA is a rare condition with an incidence of 0.4/100,000 in the UK [5]. Infection rates of IPA have increased from 0.5 cases/10,000 admissions to 6.5 cases/10,000 admissions [2]. However, IPA likely has a rate > 12 cases/year [24]. More than a thousand cases have been reported in review the literature [25, 26]. However, cases of IPA are likely underestimated as some IPAs are underdiagnosed or unreported before mortality or appeared in non-English literature.

Mycotic aortic aneurysm is a rare condition with 1–3% incidence from all aortic aneurysms [19, 27]. Stent-graft/endograft infection of the aorta is a rare clinical entity with an incidence of 0.5–3% in the conventional open surgery repair and 0.1% (6.2/1000 person years) in the endovascular repair [15, 28–31]. The incidence of aortic mycotic aneurysm resulting in IPA is 1.4% [20, 30]. In 606 patients with infective endocarditis, 4.6% has pyogenic vertebral osteomyelitis [14].

IPA associated with vascular origins, including infective endocarditis, primary aortic mycotic aneurysm, and endograft infection of aorta, has been rarely reported at an incidence of 5% [2]. The association between IPA and primary aortic mycotic aneurysm is extremely rare with a frequency of 4% [6, 20, 31, 32]. The incidence of endograft infection after endovascular repair of AAA is 0.69% in an analysis of 1302 patients [33]. IPA secondary to graft infection after aortoiliac surgery or infection of endovascular positioned stents is an uncommon clinical presentation. The incidence of IPA caused by prosthetic stent graft infection after endovascular repair of AAA is 0.39% in a study of 509 patients [29]. Acute pyogenic IPA is predominant in females [7]. Primary mycotic AAA in 6 patients (40%), stent-graft/endograft infection in 5 patients (33.3%), and infective endocarditis in 4 patients (26.7%) have been reported. IPA associated with CVD is more common in males, and observation that is consistent with a 4:1 ratio of male-to-female as found in our present study. The possible reason could be related to more cardiovascular disorders in males.

### Clinical presentation of iliopsoas abscess associated with cardiovascular disorders

Common clinical features include flank/back pain, vague abdominal pain, fever, limp, malaise, weight loss, and groin lump [5]. In a study of 61 IPA patients, features are observed with pain (95%), gastrointestinal tract complaints (43%), and lower extremity pain (30%) [13]. Three quarter (76%) of patients with IPA have pain in the abdomen, flank or back [7]. The classical clinical triad of fever, back/flank pain, and limited hip movement is present in only 30% of patients [1, 5, 8]. Half of patients with Salmonellosis of abdominal aorta have the clinical triad of fever, pulsatile abdominal mass, and back pain [23]. Clinical presentation of infected endograft of aorta includes positive blood and/or tissue culture, perigraft fluid with presence of air, and associated IPA or groin abscess on image study [15, 27, 34].

### Diagnosis of iliopsoas abscess associated with cardiovascular disorders

IPA is a relatively uncommon condition that can present itself with vague clinical presentation. Its insidious onset and occult features can delay diagnosis, resulting in high mortality and morbidity [1]. Clinical investigations of IPA should include radiology and cultures of blood, pus/tissue, and urine. IPA is commonly diagnosed via modern imaging, such as ultrasonography, CT, and magnetic resonance imaging (MRI). CT is the “gold standard” for a definitive diagnosis of IPA, although MRI can provide better discrimination of soft tissues, allowing visualization of the abscess wall and the surrounding structures. CT scan is the most sensitive and can confirm the diagnosis and define the extension of IPA [1, 3, 7, 13]. The features of IPA on CT include an enlarged IPM, with a rounded contour with contrast enhanced rim of the abscess wall, a relatively low-density area, and gas within IPM [6].

**Fig. 3:**
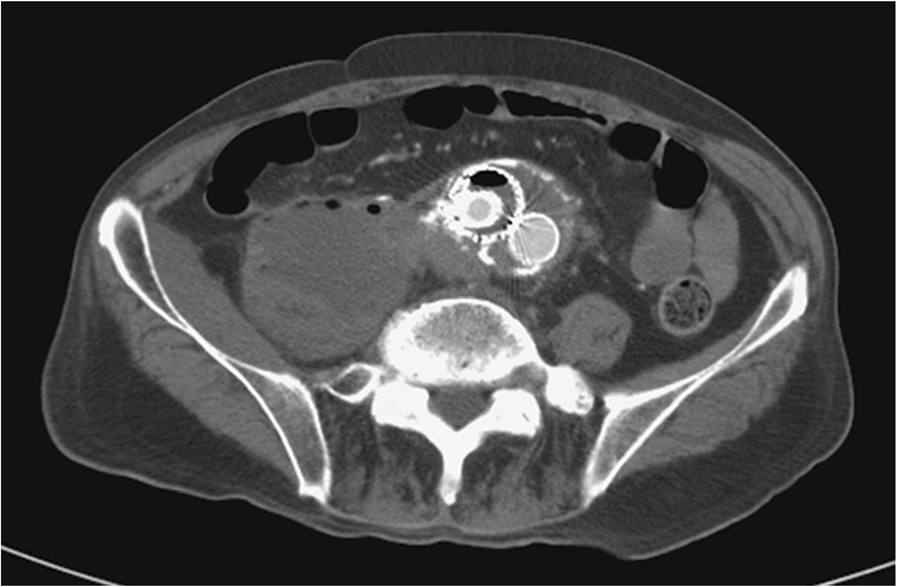
Abdominal CT depicted air within the endograft and emphysematous abscess of the right IPM

The diagnosis of mycotic aneurysm is usually depending on the clinical presentation of fever, abdominal or chest pain, positive blood cultures, and a pulsatile mass [19]. CT angiography (CTA) of the aorta or Multi-detector CT (MDCT) is the modality of choice for the vascular evaluation to detect the mycotic aneurysms and the infected endograft as an early stage. Clinical characteristics of infected aortic aneurysm or stent-graft/endograft include eccentric contour, saccular shape (especially lobulated), rapid expansion or development with periaortic/perigraft soft tissue stranding, free fluid, air pockets, and abnormal adjacent structures (vertebral body destruction, discitis, and IPA) [6, 20, 23, 27, 35, 36]. CT is the most reliable diagnostic mandatory tool for IPA related to CVD with a perfect sensitivity of 100%. Bilateral IPA in 13% patients and multiple IPAs in 25% patients are diagnosed based on CT [13]. Periodic CT scans (yearly) are recommended after endovascular repair and therefore may enable more rapid detection of gas or fluid, suggesting infection [33]. Abdominal CT depicted air within the endograft and emphysematous abscess of the right IPM in our index case of 75-year-old man with a history of an endograft implantation for AAA with an involvement of bilateral iliac arteries (Fig. 3).

Laboratory investigations are non-specific, but high levels of white blood cell count and C-reactive protein, anemia, and high erythrocyte sedimentation rates provide clinical clues of associated infection [1, 5, 20, 22].

### Microbiology of iliopsoas abscess associated with cardiovascular disorders

The most common organism in IPA with skeletal infection, regardless of primary IPA and secondary, is *Staphylococcus aureus*. In secondary IPA, most origins are from the gastrointestinal and urinary tracts, with *Escherichia coli* being the most common organism. Other organisms have also been case-reported, involving *Bacteroides* species, *Mycobacterium tuberculosis*, *Enterococcus faecalis*, *Peptostreptococcus*, Viridans group streptococci, and methicillin resistant *Staphylococcus aureus* [5]. In Taiwan, urinary tract infection (52%) of enteric microorganisms (44% of *Escherichia coli* and 24% of *Klebsiella* spp.) is the most common infection source of secondary IPA and typically found in older, female, and diabetic patients. *Klebsiella pneumoniae* has been proposed as an independent factor for IPA mortality in Taiwan [7, 8, 26].

A positive culture rate of 64.7–75% is a definitive microbial diagnosis of IPA [2, 5, 12]. Combination of blood and abscess cultures can confirm > 67% of patients with a definitive pathogen diagnosis of IPA [2]. However, blood culture can be negative for IPA in as many as 47% of patients [20]. Prior antibiotic use (inpatient antimicrobial therapy and antibiotics prior to their drainage procedure in emergency department) may have lowered the yield of culture data or may have sterilized the infection site [2].

About 5% of cultures from aortic thrombus obtained during open aneurysm repair has positive findings of Gram-positive and negative bacteria [37]. Most reported cases of IPA related to aortic mycotic aneurysm are caused by *Salmonella* infection [3]. Endovascular infection is a serious complication of non-typhoid *Salmonella* which is specifically linked to atheroscerlosis in Taiwan, particularly infrarenal abdominal aortic infected aneurysms and endocarditis [8, 38]. *Salmonella* is responsible for 27–75% of infected aortic aneurysm. *Salmonella enterica* serovar Typhimurium and *Salmonella enterica* serovar Choleraesuis are the species most commonly isolated. Other causative organisms are mycobacterium, Gram-negative bacilli other than *Salmonella*, mixed bacteria, and fungi [21, 23, 33, 39].

In aortic graft infections, the most common organisms are Gram-positive cocci (*Staphylococcus* and *Streptococcus* species are the most prevalent), although Gram-negative organisms, such as *Escherichia coli* and *Bacteroides* species, have also been isolated [40]. Only 68% of cases show a positive result in culture for a definitive microbial diagnosis of aortic graft infection. The most common culture reported in the literature is Gram-positive bacteria, with the specific bacterial organism *Staphylococcus* spp. representing 23–88% of the entire positive cultures [33]. In patients of infected aortic stent-graft/endograft, 42.1% have monomicrobial infections, and 57.9% polymicrobial infections. *Staphylococcus* (63.2%) and *Streptococcus* (31.6%) are the most common bacteria [34].

### Treatment of iliopsoas abscess associated with cardiovascular disorders

Treatment of IPA consists of application of appropriate antibiotics along with abscess drainage. For primary IPA, antistaphylococcal antibiotics should be started even before knowing the culture results. For secondary IPA, broad-spectrum antibiotics against mixed enteric bacteria should be used. Antibiotics should be properly adjusted according to the results of the abscess fluid culture and drug sensitivity. PCD through sonography/CT-guide or surgical drainage is suggested to treat IPA, especially for the primary type. PCD is less invasive and is considered the first choice. Open surgery is recommended in the case of the following: PCD failure, relative contraindication of PCD, and the presence of intraabdominal pathology which requires surgery [1, 41]. Consideration maybe given to pathogen-directed antimicrobial coverage for Gram-positive cocci in cases of primary IPA and in secondary IPA of skeletal origins, and polymicrobial coverage in cases of IPAs spreading from urinary tract or of gastrointestinal origin [2].

In the case of second stage with open definitive surgery to treat an infected AAA, endovascular stent graft should be considered as a bridge with an implantation of the endoprosthesis below the level of the bilateral renal arteries to prevent persistent aneurysmal infection [22, 27, 42]. The advantages of endovascular repair for infected AAA have reduced risks of bacterial spread and graft infections, speeding up recovery, and lowering care costs, especially for those critically sick. The cumulative rate of late conversion to open repair is 2%/year and the risk of aneurysm rupture is 1%/year [22, 31].

A combination of early PCD, debridement and resection of the surrounding tissue, aortic reconstruction with graft replacement or extra-anatomic bypass, and targeted long-term antibiotic treatment is mandatory protocol for IPA associated with CVD. The recommended duration of antibiotics use is 4–6 weeks, and in accordance with the clinical conditions [2, 8, 18, 21, 29, 32–34].

Prognosis and Mortality of iliopsoas abscess associated with cardiovascular disorders.

The mortality rate of IPA associated with CVD is 40% in our present study. Gas-formation is an important factor of clinical outcome for patients of IPA. Their mortality rate is 44.0% for the emphysematous IPA and 16.4% for the non-emphysematous IPA [41]. If *Klebsiella pneumoniae* is the microorganism of IPA, the mortality rate is 26–44%. The mortality rate is up to 100% if IPA is left untreated [7, 26, 41].

Patients of secondary IPA have longer hospital stay [3]. Patients of IPA associated with an infected aortic aneurysm have a higher incidence of emergency operation, hospital mortality, prosthetic graft infection, and aneurysm-related mortality [8]. Emphysematous IPA may be a clinical clue for ruptured mycotic aneurysm [19]. The aneurysm-related mortality rate is 57% in patients of IPA associated with aortic mycotic aneurysm. The condition is reportedly one major risk factor for patients with aortic mycotic aneurysm [39].

### Limitations of our present study on iliopsoas abscess associated with cardiovascular disorders

The major limitation of our study is its retrospective nature, small sample size and the selection of management. The infrequent nature of IPA limits our freedom to perform a prospective randomized control study. However, ours is the largest series study so far on IPA associated with CVD.

## Conclusion

Due to tis high mortality rate, clinicians should keep a high suspicion index for IPA associated with CVD through clinical presentation and physical examination. Clinicians should make a correct diagnosis via CT, CTA or MDCT to demonstrate IPA and associated infected vessels or endograft infection. Aggressive management with intervention produced outcomes better than conservative treatment with antibiotics only. Timely empiric antibiotics for common bacteria, PCD or surgical drainage for IPA, endovascular repair, or vascular reconstruction by graft replacement or bypass with intensive care are recommended mandatory to shorten hospital stay, reduce medical costs, and lower mortality rate.

## Data Availability

Readers could access the data and material supporting the conclusions of the study by contacting Sung-Yuan Hu at song9168@pie.com.tw.

## Abbreviations

AAA: Abdominal aortic aneurysm
CT: Computed tomographic
CTA: Computed tomographic angiography
CVD: Cardiovascular disorders
DM: Diabetes mellitus
ICU: Intensive care unit
IPA: Iliopsoas abscess
IPM: Iliopsoas muscle
MDCT: Multi-detector computed tomographic
MRI: Magnetic resonance imaging
PCD: Percutaneous drainage
TEE: Transesophageal echocardiography
